# Enhancement of chemotherapy and radiotherapy of murine tumours by AQ4N, a bioreductively activated anti-tumour agent

**DOI:** 10.1054/bjoc.2000.1163

**Published:** 2000-05-18

**Authors:** L H Patterson, S R McKeown, K Ruparelia, J A Double, M C Bibby, S Cole, I J Stratford

**Affiliations:** School of Pharmacy and Pharmaceutical Sciences, De Montfort University, Leicester, LE1 9BH, UK; School of Biomedical Sciences, University of Ulster, Jordanstown, Newtownabbey BT37 OQ, Northern Ireland; Clinical Oncology Unit, University of Bradford, Bradford, BD7 1DP, UK; MRC Radiation and Genome Stability Unit, Harwell, Oxon, OX11 ORD, UK; School of Pharmacy and Pharmaceutical Sciences, University of Manchester, Manchester, M13 9PL, UK

**Keywords:** AQ4N, radiotherapy, bioreduction, cyclophosphamide, cisplatin, thiotepa

## Abstract

AQ4 (1,4-Bis-{[2-(dimethylamino-N-oxide)ethyl]amino}5,8-dihydroxyanthracene-9, 10-dione) is a prodrug designed to be excluded from cell nuclei until bioreduced in hypoxic cells to AQ4, a DNA intercalator and topoisomerase II poison. Thus, AQ4N is a highly selective bioreductive drug that is activated in, and is preferentially toxic to, hypoxic cells in tumours. Five murine tumours (MAC16, MAC26, NT, SCCVII and RIF-1) have been used to investigate the anti-tumour effects of AQ4N. In only one tumour (MAC16) was AQ4N shown to be active as a single agent. However, when combined with methods to increase the hypoxic tumour fraction in RIF-1 (by physical clamping) and MAC26 tumours (using hydralazine) there was a substantial enhancement in anti-tumour effect. Notably, RIF-1 tumours treated with AQ4N (250 mg kg^−1^) followed 15 min later by physically occluding the blood supply to the tumour for 90 min, resulted in a 13-fold increase in growth delay. When combined with radiation or chemotherapy, AQ4N substantially increased the effectiveness of these modalities in a range of in vivo model systems. AQ4N potentiates the action of radiation in both a drug and radiation dose-dependent manner. Further the enhancement observed is schedule-independent with AQ4N giving similar effects when given at any time within 16 h before or after the radiation treatment. In combination with chemotherapy it is shown that AQ4N potentiates the activity of cyclophosphamide, cisplatin and thiotepa. Both the chemotherapeutic drugs and AQ4N are given at doses which individually are close to their estimated maximum tolerated dose (data not included) which provides indirect evidence that in the combination chemotherapy experiments there is some tumour selectivity in the enhanced action of the drugs. © 2000 Cancer Research Campaign


*British Journal of Cancer* (2000) **82**(12), 1984–1990
© 2000 Cancer Research Campaign
doi: 10.1054/ bjoc.2000.1163, available online at http://www.idealibrary.com on IDEAL®

### Enhancement of chemotherapy and radiotherapy of murine tumours by AQ4N, a bioreductively activated anti-tumour agent

**LH Patterson[1], SR McKeown[2], K Ruparelia[1], JA Double[3], MC Bibby[3], S Cole[4] and IJ Stratford[4,5]**

[1]School of Pharmacy and Pharmaceutical Sciences, De Montfort University, Leicester LE1 9BH, UK; [2]School of Biomedical Sciences, University of Ulster, Jordanstown, Newtownabbey BT37 OQ, Northern Ireland; [3]Clinical Oncology Unit, University of Bradford, Bradford BD7 1DP, UK; [4]MRC Radiation and Genome Stability Unit, Harwell, Oxon OX11 ORD, UK; [5]School of Pharmacy and Pharmaceutical Sciences, University of Manchester, Manchester M13 9PL, UK

**Summary** AQ4 (1,4-Bis-{[2-(dimethylamino-N-oxide)ethyl]amino}5,8-dihydroxyanthracene-9, 10-dione) is a prodrug designed to be excluded from cell nuclei until bioreduced in hypoxic cells to AQ4, a DNA intercalator and topoisomerase II poison. Thus, AQ4N is a highly selective bioreductive drug that is activated in, and is preferentially toxic to, hypoxic cells in tumours. Five murine tumours (MAC16, MAC26, NT, SCCVII and RIF-1) have been used to investigate the anti-tumour effects of AQ4N. In only one tumour (MAC16) was AQ4N shown to be active as a single agent. However, when combined with methods to increase the hypoxic tumour fraction in RIF-1 (by physical clamping) and MAC26 tumours (using hydralazine) there was a substantial enhancement in anti-tumour effect. Notably, RIF-1 tumours treated with AQ4N (250 mg $kg^{-1}$) followed 15 min later by physically occluding the blood supply to the tumour for 90 min, resulted in a 13-fold increase in growth delay. When combined with radiation or chemotherapy, AQ4N substantially increased the effectiveness of these modalities in a range of in vivo model systems. AQ4N potentiates the action of radiation in both a drug and radiation dose-dependent manner. Further the enhancement observed is schedule-independent with AQ4N giving similar effects when given at any time within 16 h before or after the radiation treatment. In combination with chemotherapy it is shown that AQ4N potentiates the activity of cyclophosphamide, cisplatin and thiotepa. Both the chemotherapeutic drugs and AQ4N are given at doses which individually are close to their estimated maximum tolerated dose (data not included) which provides indirect evidence that in the combination chemotherapy experiments there is some tumour selectivity in the enhanced action of the drugs. © 2000 Cancer Research Campaign

**Keywords:** AQ4N; radiotherapy; bioreduction; cyclophosphamide; cisplatin; thiotepa

Hypoxia occurs in regions of solid tumours as a result of poorly formed micro-vasculature coupled with the metabolic demand of tumour cells close to the available blood supply; this compromises the oxygen availability to more distant tumour cells (Brown and Giaccia, 1994). Hypoxic regions are known to be resistant to radiotherapy and conventional chemotherapy and this has led to much interest in the discovery of cytotoxins that kill hypoxic tumour cells (reviewed in Stratford and Workman, 1998). These agents are designed to be bioreduced selectively in cells low in oxygen to produce potent cytotoxins. Synthetic compounds with chemotherapeutic potential as bioreductive agents are usually based on nitroaromatic or quinone structures, e.g. nitroimidazoles and mitosenes (indoloquinones) (Workman and Stratford, 1993; Denny et al, 1996; Patterson and Raleigh, 1998). These agents are cytotoxic, on reduction, due to their ability to covalently bind to DNA that will not occur in oxygenated cells. A benzotriazene di-N-oxide, tirapazamine, is currently the most promising bioreductive drug and is in phase II/III clinical trials. This agent can act as a chemosensitizer and radiation enhancer due primarily to toxicity of the free radical species formed transiently during its reduction to the non-toxic reduction product SR4317. The toxic effect is probably caused by direct local damage to DNA that results in DNA strand-breaks and chromosome breaks that are difficult to repair (Brown and Wang, 1998).

To date, therefore, bioreductive agents generate transient reactive species that are entirely dependent on hypoxia to generate the toxic species. In contrast, we report here preclinical data on a new class of bioreductive agent exemplified by AQ4N (1,4-bis-{[2-(dimethylamino-N-oxide)ethyl]amino}5,8-dihydroxyanthracene-9, 10-dione). This agent is a prodrug designed to be excluded from cell nuclei (Patterson, 1993; Smith et al, 1997*b*) until metabolized in hypoxic cells to give AQ4, a stable, oxygen-insensitive metabolite (Smith et al, 1997*a*) (Figure 1). AQ4 is a DNA intercalator and potent inhibitor of DNA type II topoisomerase (Patterson, 1993; Patterson et al, 1994; Smith et al, 1997*a*). Previously we have shown that AQ4N is an effective enhancer of the anti-tumour effect of radiation in one murine tumour model, i.e. the T50/80 mammary carcinoma grown in BDF mice (McKeown et al, 1995, 1996). We now report on the efficacy of this drug in several murine models. These models were selected as they have previously been used successfully to show the efficacy of other bioreductive drugs when used in combination with radiation or chemotherapeutic agents (Stratford et al, 1989; Bremner et al, 1990; Bibby et al, 1989).

*Received 1 October 1999*
*Revised 11 February 2000*
*Accepted 17 February 2000*

*Correspondence to:* LH Patterson, The School of Pharmacy, University of London, 29/39 Brunswick Square, London WC1N 1AX, UK

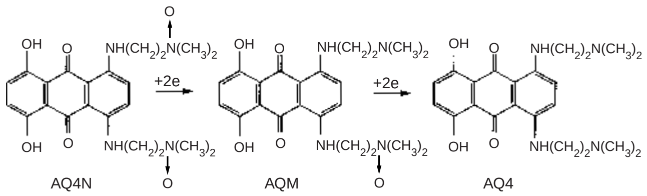

**Figure 1** AQ4N and its reduction metabolities

### MATERIALS AND METHODS

### Materials

AQ4N and 1,4-bis-{[2-(dimethylamino)ethyl]amino}5,8-dihydroxy-anthracene-9,10-dione (AQ4) were synthesized as described previously (Patterson et al, 1994). Cisplatin, cyclophosphamide, thiotepa and hydralazine were obtained from commercial sources. Materials for cell culture were from Flow Labs (Irvine, UK) and all other chemicals were used as purchased from Sigma (Poole, Dorset, UK).

### Murine tumour models

*MAC 16, MAC26, RIF-1, NT and SCCV11 tumour-bearing animals*

Pure strain NMRI male mice, aged 6–8 weeks were used for experiments with MAC16 and MAC26 tumours (Double and Ball, 1975; Bibby et al, 1987). Male C3H mice (8–12 weeks) were used for experiments using the RIF-1 and SCCVII tumours. NT tumours were grown in male CBA mice (Denekamp and Harris, 1975). RIF-1 and SCCVII tumours were maintained in mice by in vivo–in vitro passage as described previously by Twentyman et al (1980). Tumours were passaged using tumour brei from a donor mouse or by implantation of fragments. Tumours for experiments were implanted subcutaneously in the mid dorsal region of the back. All animal procedures were carried out under project licences issued by the Home Office, London, and UKCCCR guidelines (Workman et al, 1988) for the use of animals in experiments were adhered to throughout.

*Tumour systems and treatments*

***Radiation treatments*** To allow local irradiation of tumours, unanaesthetized mice were gently restrained in polyvinyl jigs with lead shielding and a cut-away section over the tumour. Irradiations were done with 250 kV X-rays as described by Stratford et al (1989). In most experiments, single high doses of radiation were used (20 Gy for the SCCVII tumour; 25 Gy for the RIF-1 tumour, 12 Gy for the NT tumour). These doses were chosen to kill most of the oxic cells in these tumours, thereby creating tumours which contained predominantly radio-resistant hypoxic cells. This mode of treatment should reveal the cytotoxicity of bioreductive drugs selectively toxic to hypoxic cells when injected into mice after local irradiation of the tumours.

***Drug treatments*** AQ4N was usually given at its maximum tolerated single dose (MTD) which was 200–250 mg kg$^{-1}$ (data not shown). However, in drug combination experiments, toxicity was observed with some chemotherapy agents, most notably cisplatin. The doses used were therefore at or close to the MTD of the combination as determined from pilot experiments (data not shown). All drugs were dissolved in phosphate-buffered saline (PBS) and administered to mice by the intraperitoneal (i.p.) route in a 0.1 ml injection volume.

*Induction of hypoxia in tumours*

Two methods were employed to temporarily reduce the oxygen status of the tumours. The first of these was physically to occlude the blood supply to the tumours by applying a hinged, metal D-shaped clamp around the base of the tumour to occlude the vasculature for 90 min (Denekemp et al, 1983). The second was to treat mice with 10 mg kg$^{-1}$ hydralazine. Both these methods can induce 100% radiobiological hypoxia in experimental tumours (Acker and Chaplin, 1987). In these experiments, mice were treated with AQ4N, then 15 min later, when it is presumed that tumour levels of drug will be high, the tumours were clamped or mice treated with hydralazine.

### Measurement of tumour response to treatment

*Tumour growth delay*

For NT tumours, treatment was initiated when the tumour reached 6.5–7.5 mm GMD (geometric mean of three orthogonal diameters). Tumours were measured three times weekly and the time taken to reach double its treatment volume was used as a measure of antitumour efficacy. For RIF-1 tumours, treatment was initiated when the tumours reached 100–200 mm$^3$ and their growth followed until they had reached 4 × the treatment volume. MAC16 and MAC26 tumours were treated when the two longest orthogonal diameters were at least 4 and 5 mm. Tumour response was measured twice weekly by two-dimensional caliper measurements. For RIF-1, MAC16 and MAC26, tumour growth delay was calculated by subtracting the mean time to grow to 4 × treatment volume for control tumours from that obtained after drug exposure.

*In vivo–in vitro clonogenic assay*

C3H mice bearing SCCVII or RIF-1 tumours were treated upon reaching a volume of 100–200 mm$^3$. After 24 h tumours were excised, minced and digested with an enzyme cocktail (6 mg DNase, 2 mg collagenase and 2 mg pronase per 10 ml PBS) to produce a single cell suspension. The cells were counted, diluted as necessary, plated and incubated at 37°C in 95% air/5% carbon dioxide for 12–14 days. Colonies were fixed, stained with 0.4% crystal violet in methanol and scored by eye. Surviving fraction (SF) was calculated as the number of colonies counted divided by the number of cells plated for a given treatment, divided by the same fraction determined for untreated control tumours.

### RESULTS

### Activity of AQ4N when combined with methods to induce hypoxia

To investigate the effect of tumour hypoxia on AQ4N activity a physical clamp and a vascular 'steal' agent, hydralazine were used. Table 1 shows that AQ4N, clamping or hydralazine alone does not significantly delay tumour growth in RIF-1 or MAC26 tumours. However, making the tumours completely hypoxic for 90 min, using a physical clamp, after AQ4N administration does significantly delay tumour growth by 13-fold. Hydralazine, which

© 2000 Cancer Research Campaign

**Table 1** Activity of AQ4N in combination with hypoxia inducing protocols

| Treatment | Tumour model | Growth delay[a] (days) | *n* |
|---|---|---|---|
| Hypoxia | | | |
| AQ4N | RIF-1 | 0.6±0.5 | 5 |
| Clamp alone | RIF-1 | 1.1±0.9 | 14 |
| AQ4N + clamp | RIF-1 | 14.1±1.7 | 8 |
| AQ4N | MAC26 | 0 | 5 |
| AQ4N + hydralazine | MAC26 | 5.5* | 5 |
| Hydralazine | MAC26 | 0 | 5 |
| Radiation | | | |
| Radiation (25 Gy) | RIF-1 | 31.1±2.0 | 19 |
| Radiation (25 Gy) + AQ4N (AQ4N immediately after radiation) | RIF-1 | 59.7±4.8 | 5 |
| AQ4N | NT | 0.05±0.6 | 8 |
| Radiation (12Gy) | NT | 3.76±2.1 | 8 |
| AQ4N (AQ4N 30 min before radiation) + Radation (12 Gy) | NT | 13.76±3.8** | 8 |

Growth delay = time to reach 4 × tumour volume at treatment (treated group minus untreated group) for RIF -1 and MAC26; NT tumours were followed to 2 × treatment volume. AQ4N dosed i.p. MAC tumours: 50 mg $kg^{-1}$; Hydralazine (10 mg $kg^{-1}$) was given 10 min after AQ4N, RIF-1 and NT tumours: 200 mg $kg^{-1}$; clamping for 90 min was carried out 15 min after AQ4N administration. See Materials and Methods for experimental details. Mann–Whitney *U*-test: * $P < 0.05$, **$P < 0.01$.

**Table 2** Activity of AQ4N in combination chemotherapy against murine tumours in vivo

| Treatment and Time schedule in minutes | Drug dose (mg $kg^{-1}$) | Tumour | Growth delay[a] (days) | Surviving fractions[b] |
|---|---|---|---|---|
| AQ4N | 50 | MAC16 | 4.6 | – |
| Thiotepa | 10 | MAC16 | 3.7 | – |
| AQ4N 1 min + thiotepa | 50/10 | MAC1615.2** | – | |
| AQ4N | 200 | RIF-1 | 0.5 | – |
| CPM | 50 | RIF-1 | 0.1 | – |
| AQ4N 60 min + CPM | 200/50 | RIF-1 | 11.4*** | – |
| AQ4N | 250 | SCCVII | – | 1.4 |
| CPM | 100 | SCCVII | – | 0.0052 |
| AQ4N 60 min + CPM | 250/100 | SCCVII | – | <0.00001 |
| AQ4N | 100 | RIF-1 | – | 0.7 |
| Cisplatin | 8 | RIF-1 | – | 0.00023 |
| AQ4N 30 min + cisplatin | 100/8 | RIf-1 | – | <0.000001 |
| AQ4N | 100 | SCCVII | – | 0.77 |
| Cisplatin | 8 | SCCVII | – | 0.0004 |
| AQ4N 30 min + cisplatin | 100/8 | SCCVII | – | <0.000001 |

[a] Time to 4 × initial tumour volume (treated group minus untreated group). [b]Surviving fraction of clonogenic cells following treatment. All drugs were dosed i.p. See Materials and Methods for experimental details. CPM = cyclophosphamide. All results ($n \geq 5$ per treatment group). Mann-Whitney U-test: *$P < 0.05$, **$P < 0.01$, ***$P < 0.001$.

acts as a partial pharmacological clamp also, increases the anti-tumour effect of AQ4N in MAC26 tumours.

### *Activity of AQ4N in combination chemotherapy*

Table 2 shows that when AQ4N (50 mg $kg^{-1}$) or thiotepa (10 mg $kg^{-1}$) alone were tested for anti-tumour efficacy against the MAC16 tumour only a small effect (approx. 4 days delay) on tumour growth was seen; combination of AQ4N and thiotepa at these concentrations significantly delayed tumour growth by over 15 days ($P < 0.01$). Table 2 also shows that against RIF-1 tumours, a combination of AQ4N (200 mg $kg^{-1}$) and cyclophosphamide (50 mg $kg^{-1}$) significantly delays tumour growth whereas, as single agents, neither drug has a measurable effect. Similar results are seen with SCCVII where clonogenic cell survival was below the limit of detection following a combination of AQ4N (250 mg $kg^{-1}$) and cyclophosphamide (100 mg $kg^{-1}$). Scheduling of drug administration is important since tumour regression (in RIF-1) and clonogenic cell kill (in SCCVII) is greater when cyclophosphamide is administered 1 h prior to AQ4N compared to 1 h following AQ4N (results not shown). In both the RIF-1 and SCCVII models the anti-tumour effect of 8 mg $kg^{-1}$ cisplatin was significantly enhanced by combination with 100 mg $kg^{-1}$ AQ4N (Table 2).

 *© 2000 Cancer Research Campaign*

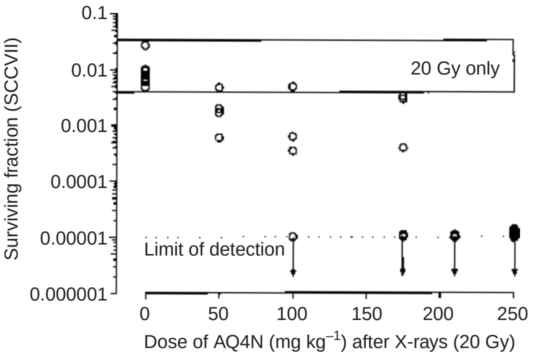

**Figure 2** The effect on surviving fraction of SCCVII tumours of various doses of AQ4N after 20 Gy of X-radiation. C3H mice bearing the SCCVII tumour were treated (five per group) with a range of AQ4N doses 30 min after exposure to 20 Gy X-radiation. Mice were sacrificed 24 h later, the tumour excised and the number of clonogenic cells determined as described in Materials and Methods. Results are given for individual tumours. Those which showed no clonogenic cells within the limits of detection of the assay are indicated with downward pointing arrows

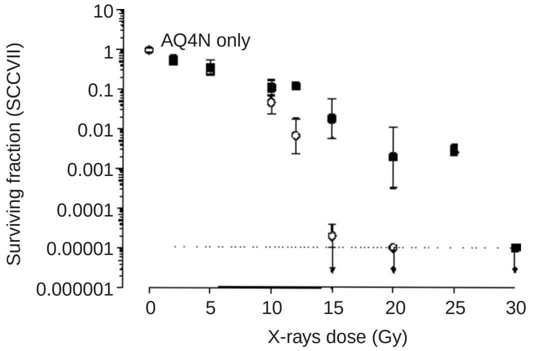

**Figure 3** The effect on surviving fraction of SCCVII tumours of 250 mg kg$^{-1}$ of AQ4N after various doses of X-radiation. C3H mice bearing the SCCVII tumour were treated with a range of X-radiation doses with (open circles) or without (closed squares) 250 mg kg$^{-1}$ of AQ4N. Drug was administered 30 min after exposure to radiation. Mice were sacrificed 24 h later, the tumour excised and the number of clonogenic cells determined as described in Materials and Methods. Results are the means ± s.e.m. $n = 5$. Tumours which showed no clongenic cells within the limits of detection of the assay are indicated with downward pointing arrows

### *AQ4N in combination with ionizing irradiation*

Previously we have shown (McKeown et al, 1995, 1996) that in the T50/80 tumour AQ4N enhances the effect of radiation. This was confirmed in the RIF-1 and NT tumour models (Table 1) and led to a more detailed study in the SCCVII model (Figures 2, 3 and 4). Figure 2 shows that AQ4N considerably enhances the clonogenic cell kill produced by a single dose of ionizing radiation (20 Gy) over a range of AQ4N concentrations (100–250 mg kg$^{-1}$). In fact, AQ4N doses greater than 200 mg kg$^{-1}$ in combination with ionizing radiation were so effective that the number of surviving clonogenic cells was below the limit of detection. At this dose of drug alone there was little, if any, measurable killing of tumour cells.

We also investigated the effect of a range of radiation doses (0–30 Gy) immediately followed by a single dose of AQ4N (250 mg kg$^{-1}$) in C3H mice bearing SCCVII tumours (Figure 3). There was a marked decrease in clonogenic cell survival as compared to irradiation alone especially at radiation doses above

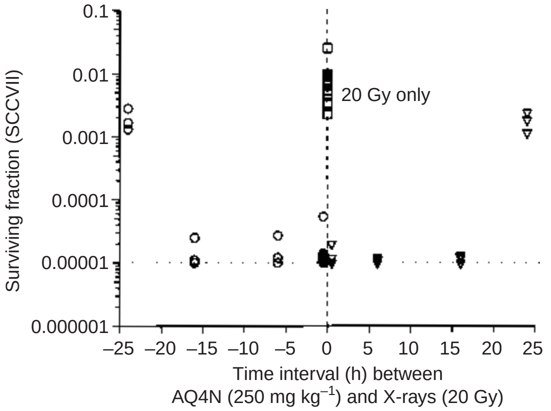

**Figure 4** The effect on surviving fraction of SCCVII tumours of dosing AQ4N at different times before or after X-radiation. C3H mice bearing the SCCVII tumour (at least three animals per treatment group) were irradiated with 20 Gy X-radiation. They were also administered with a single i.p. dose of AQ4N (250 mg kg$^{-1}$) at known times intervals up to 24 h before (o) or after radiation (▽). Mice were sacrificed 24 h after each dosing interval, the tumour excised and the number of clonogenic cells determined as described in Materials and Methods. Results are given for individual tumours

10 Gy. This will be because, at doses of 10 Gy and below, the response of the tumour will be dominated by the aerobic population of tumour cells, i.e. killing of the radiation-resistant hypoxic cells will only be revealed with single doses of radiation > 10 Gy. Since the level of tumour cell killing following 15 Gy plus AQ4N is only seen when radiation alone is given at 30 Gy, we can calculate a dose modification of 2.0 at this level of cell kill. The effect of administering AQ4N at various times before or after irradiation of SCCVII tumour-bearing mice was subsequently investigated (see Figure 4). This shows that i.p. injection of AQ4N (250 mg kg$^{-1}$) up to 16 h before irradiation or up to 16 h following irradiation (20 Gy) produced a substantial increase in cell kill when these intervals were increased to 24 h the potentiating effect is lost.

### DISCUSSION

The rationale underlying the development of AQ4N was to design a drug with potential for targeting DNA and DNA associated enzymes, but which was not active until metabolized in hypoxic tumour cells. The DNA affinity of several clinically useful agents, including the anthracyclines and mitoxantrone, is associated with a planar, electron-deficient chromophore bearing cationic amino side arm(s). It was proposed that an N-oxide of a tertiary aliphatic amine is electrically neutral and thus will exhibit a markedly decreased DNA binding affinity compared to the reduced cationic tertiary amine (Patterson, 1993). In fact previous studies have shown that AQ4 has excellent DNA binding affinity (Patterson et al, 1994) and is a DNA type II topoisomerase inhibitor (Smith et al, 1997*a*). In contrast AQ4N had a equilibrium binding to DNA which was too low to measure and AQ4N exhibited a 50-fold decrease in topoisomerase II inhibitory activity as compared to AQ4. AQ4N, in combination with hypoxia was a very poor cytotoxin against rodent and human cell lines whilst its reduction product, AQ4 is a potent cytotoxic agent (Smith et al, 1997*a*). This was confirmed in several other cell lines (Wilson et al, 1996).

Previously using the T50/80 murine tumour model in vivo we have observed potent anti-tumour activity of AQ4N when used in

*© 2000 Cancer Research Campaign*

combination with radiation (McKeown et al, 1995, 1996). For all bioreductive drugs to be useful clinically, tumour expression in vivo of appropriate reductases will be an important determinant of efficacy. Although not addressed in this study AQ4N is known to be reductively metabolized by haem containing enzymes including cytochrome P450s (CYPs). Specifically, AQ4N is a substrate for the CYP3A subfamily under hypoxic conditions (Raleigh et al, 1998) and CYP3A has been measured in RIF-1 and SCCVII tumours (Murray et al, 1998). CYP3A isoforms have been detected in a broad spectrum of human cancers including colon (Massaad et al, 1993; McKay et al, 1993) breast (Murray et al, 1993) and lung (Kivisto et al, 1995). Unlike tirapazamine it has not proved possible to metabolize AQ4N in cultured tumour cells, this is almost certainly due to the fact that cytochome P450s are known to be down-regulated ex vivo (Patterson et al, 1998). We have previously shown that the potential to metabolize AQ4N disappears within 24 h of excision in the T50/80 tumour (Hejmadi et al, 1996). The present studies were undertaken to further examine the potential of AQ4N to enhance radiation and chemotherapy in a number of in vivo murine tumours and hence support its use as a hypoxia activated bioreductive agent in the clinic.

### Enhancement of AQ4N metabolism in vivo by hypoxia

Evidence that AQ4N is acting as a hypoxia-activated cytotoxin comes from the combination of this agent with clamping or hydralazine (Table 1). Clearly, the production of 100% hypoxia with a tumour clamp enhances the anti-tumour effect of AQ4N administered as a single agent and supports the contention that AQ4N can be bioreductively activated in vivo. Enhancement of the anti-tumour activity of bioreductive drugs with the vasoactive agent, hydralazine, has previously been shown with RSU 1069 (Acker and Chaplin, 1987; Stratford et al, 1997; Brown et al, 1987) and mitomycin-C (Adams et al, 1989; Quinn et al, 1992). It is known that the treatment of mice bearing the MAC26 colon tumour with hydralazine produces 80% shut down in functional tumour vasculature and greater than 60% reduction in tumour blood perfusion (Quinn et al, 1992). The present results show that hydralazine significantly enhances the anti-tumour efficacy of AQ4N again supporting the hypothesis that it is bioreductively activated in vivo despite poor activation in vitro. Our interpretation of the present data supports other published studies. Notably, AQ4N was active against the mouse mammary tumour MDAH-MCa-4 when combined with the tumour blood flow inhibitor 5, 6-dimethylxanthenone-4-acetic acid (DMXAA) (Wilson et al, 1996). In addition, the anti-tumour effect of AQ4N in vivo was potentiated in T50/80 tumours by combination with hypobaric hypoxia with a dose enhancement ratio of 5.1 (McKeown et al, 1995).

### Enhancement of chemotherapy by AQ4N

From the results presented in Table 2 it can clearly be seen that AQ4N significantly enhances the effect of three standard chemotherapy agents (cisplatin, cyclophosphamide and thiotepa). This supports previous results that showed that AQ4N enhances the anti-tumour effect of cyclophosphamide and cisplatin in the T50/80 tumour (McKeown et al, 1998). The results with the RIF-1 and SCCVII model systems are consistent with the chemotherapeutic enhancement effects of other bioreductive agents, including tirapazamine (Kim and Brown, 1994; Siim et al, 1997) and E09 (Bibby et al, 1993) in these tumour types. MAC16 is an unusual model since it is poorly responsive to most conventional anticancer drugs (Bibby et al, 1988). This may be in part because MAC16 is a relatively slow growing tumour becoming necrotic as it grows suggesting the presence of a significant level of hypoxia. The significantly enhanced effect of thiotepa in combination with AQ4N suggests that this approach might have particular value in control of colon tumours.

Standard chemotherapy drugs typically target the well-perfused and hence oxygenated tumour regions. The enhancement by AQ4N on the anti-tumour effects of these drugs may be explained by the existence in the hypoxic regions of AQ4 which would exert its cytotoxicity on cells as they attempt to re-enter the cell cycle, in response to the damage accrued by the well oxygenated cells. Recently, AQ4N over a range of concentrations has been shown to enhance the anti-tumour effects of cyclophosphamide in T50/80 implants. In particular, the anti-tumour effect of AQ4N (100 mg $kg^{-1}$) combined with cyclophosphamide (100 mg $kg^{-1}$) was shown to be equivalent to that produced by a single 200 mg $kg^{-1}$ dose of cyclophosphamide alone, i.e. a 50% reduction in cyclophosphamide dose (McKeown et al, 1998).

### Enhancement of radiation therapy by AQ4N

Combination of AQ4N (200 mg $kg^{-1}$) with ionizing radiation (25 Gy) slowed RIF-1 tumour growth by over 40% compared to radiation alone (Table 1). This encouraging result was explored more thoroughly using SCCVII implants, which showed that AQ4N, over a range of concentrations, greatly enhanced the positive effects of radiation (20 Gy). When AQ4N and radiation are administered at approximately the same time, a positive interaction can be explained on the basis that the two modalities are cytotoxic to different (i.e. oxic or hypoxic) cell populations. However, with AQ4N the observed enhancement is maintained even when drug is administered up to 16 h before radiation in the SCCVII tumour model (Figure 4); the stability and DNA affinity of AQ4 is likely to explain this phenomenon (Smith et al, 1997*a*; 1997*b*). Once produced, AQ4 will persist in the hypoxic cell fraction and kill any cell which attempts to re-enter the cell cycle following destruction of oxic cells by irradiation. When AQ4N was administered up to 16 h after irradiation, an enhancement was also observed. This suggests that formation of AQ4 in hypoxic cells that have not yet re-oxygenated, can still prevent them from repopulating the tumour. In T50/80 tumours a similar, although even longer, positive interaction time was observed with a 50% dose-sparing effect reported for up to 4 days before and 6 h after irradiation (Hejmadi et al, 1996; McKeown et al, 1996). An enhanced anti-tumour activity post radiation was also observed in MDAH-Mca-4 tumours (Wilson et al, 1996). Overall this and previous studies are consistent with AQ4N acting as a bioreductive radiation enhancer. Differences in the time scales of the radiation enhancement by AQ4N in SCCVII compared to T50/80 tumours may be related to tumour clearance and/or host animal clearance of compound. The ability of cells to survive in the hypoxic compartment of tumours may also be a factor. Moore (1988) has reported that T50/80 tumours have a particularly high hypoxic fraction (60%) which suggests that T50/80 cells are capable of remaining viable, when hypoxic, for some considerable time; a

© 2000 Cancer Research Campaign factor which might also account for the very long times of interaction in this model previously observed (McKeown et al, 1995). In contrast, SCCVII tumours are significantly better oxygenated and have a faster doubling time than T50/80. This may explain the shorter time of maximal interaction, i.e. 16 h versus 96 h in the T50/80 tumor. The stable DNA-affinic agent AQ4, which is produced in the hypoxic cells, will compromise the cells in which it is generated for as long as they are viable. Once these cells die and become necrotic the cells, and the associated AQ4, are no longer capable of influencing tumour responses. This is likely to occur in fast growing tumours (e.g. SCCVII) more quickly than slow growing tumours especially those with a high proportion of hypoxic cells (e.g. T50/80).

In conclusion AQ4N acts like a bioreductive drug when combined with hypoxia, chemotherapy or radiation treatment in vivo. The efficacy of the drug has now been shown against MAC16, MAC 26, NT, RIF-1 and SCCVII, T50/80 (McKeown et al, 1995, 1996) and MDAH-Mca-4 (Wilson et al, 1996). They all support the use of AQ4N as a bioreductive agent that has little or no intrinsic activity per se but is a very efficient enhancer of radiation as well as standard chemotherapy agents such as cyclophosphamide, cisplatin and thiotepa. An additional benefit of this bioreductively activated cytotoxin is the stable nature of the reduction product, AQ4, which may be capable of eliciting a bystander cell killing effect on proximate non-hypoxic tumour regions. In support of this confocal microscopy shows that viable cells preloaded with AQ4, the authentic reduction product of AQ4N liberate drug within 2 h despite the high DNA affinity of the latter (Smith et al, 1997*b*).

### REFERENCES

Acker B and Chaplin D (1987) Potentiation of tumor-cytotoxicity of RSU-1069 by hydralazine: a new approach toward overcoming hypoxic radioresistance. *Clin Invest Med* **10**: B114

Adams GE, Stratford IJ and Nethersell ABW (1989) Manipulation of the oxygenation of tumours: Activation of bioreductive drugs. *Br J Radiol BIR Rep* **19**: 71–75

Bibby MC, Double JA, Ali SA, Fearon KCH, Brennan RA and Tisdale MJ (1987) Characterization of a transplantable adenocarcinoma of the mouse colon producing cachexia in recipient animals. *J Natl Cancer Inst* **78**: 539–546

Bibby MC, Double JA and Loadman PM (1988) Unique chemosensitivity of MAC16 tumors to flavone acetic-acid (Lm975, Nsc-347512). *Br J Cancer* **58**: 341–344

Bibby MC, Cronin BP and Phillips RM (1993) Evaluation of the cytotoxicity of the indoloquinone Eo9 in a human colon adenocarcinoma model. *Intl J Oncol* **3**: 661–666

Bremner JCM, Startford IJ, Bowler J and Adams GE (1990) Bioreductive drugs and the selective induction of tumour hypoxia. *Br J Cancer* **61**: 717–721

Brown JM and Giaccia AJ (1994) Tumor hypoxia – the picture has changed in the 1990s. *Int J Radiat Biol* **65**: 95–102

Brown JM and Wang L.-H (1998) Tirapazamine: laboratory data relevant to clinical activity. *Anti-Cancer Drug Design* **13**: 529–539

Brown JM, Zeman EM, Lemmon M and Hirst KV (1987) Selective antitumor therapy with a new class of bioreductive agents. *Proc Am Assoc Cancer Res* **28**: 310

Denekamp J and Harris SR (1975) Tests of two electron affinic sensitizers in vivo using regrowth of an experimental carcinoma. *Radiat Res* **61**: 191–197

Denekemp J, Hill S and Hobson B (1983) Vascular occlusion and tumour cell death. *Eur J Cancer Clin Oncol* **19**: 271–279

Denny WA, Wilson WR and Hay MP (1996) Recent developments in the design of bioreductive drugs *Br J Cancer* **74**: S32–S38

Double JA and Ball CR (1975) Chemotherapy of transplantable adenocarcinomas of the colon in mice. *Cancer Chemother Rep* **59**: 1083–1089

Hejmadi MV, McKeown SR, Friery OP, McIntyre IA, Patterson LH and Hirst DG (1996) DNA damage following combination of radiation with the bioreductive drug AQ4N: Possible selective toxicity to oxic and hypoxic tumour cells. *Br J Cancer* **73**: 499–505

Kim IH and Brown JM (1994) Rexoygenation and rehypoxiation in the SCCVII mouse-tumor. *Int J Radiat Oncol Biol Physics*, **29**: 493–497

Kivisto KT, Fritz P, Linder A, Friedel G, Beaune P and Kroemer HK (1995) Immunohistochemical localization of cytochrome-P450 3A in human pulmonary carcinomas and normal bronchial tissue. *Histochem Cell Biol* **103**: 25–29

McKay JA, Murray GI, Weaver RJ, Ewen SWB, Melvin WT and Burke MD (1993) Xenobiotic-metabolizing enzyme expression in colonic neoplasia. *Gut* **34**: 1234–1239

McKeown SR, Hejmadi MV, McIntyre IA, McAleer JJA and Patterson LH (1995) AQ4N – an alkylaminoanthraquinone N-oxide showing bioreductive potential and positive interaction with radiation in vivo. *Br J Cancer* **72**: 76–81

McKeown SR, Friery OP, McIntyre IA, Hejmadi MV, Patterson LH and Hirst DG (1996) Evidence for a therapeutic gain when AQ4N or tirapazamine is combined with radiation. *Br J Cancer* **74**: S39–S42

McKeown SR, Friery O, Gallagher R, Murray M, Clarke C, Patterson LH and Hirst DG (1998) Enhancement of cyclophosphamide anti-tumour activity with bioreductive drugs AQ4N and tirapazamine. *Br J Cancer* **78**: 45

Massaad L, Dewaziers I, Ribrag V, Janot F, Morizet J, Beaune PH, Gouyette A and Chabot GG (1993) Screening of main drug-metabolizing-enzymes in mouse and human colon tumours. *Bull Cancer* **80**: 397–407

Moore JV (1988) The dynamics of tumour cords in an irradiated mouse mammary carcinoma with a large hypoxic cell component. *Jpn J Cancer Res (Gann)* **79**: 236–243

Murray GI, Weaver RJ, Paterson PJ, Ewen SWB, Melvin WT and Burke MD (1993) Expression of xenobiotic metabolizing enzymes in breast cancer. *J Pathology* **169**: 347–353

Murray MM, Robson T, Gallagher R, Patterson LH, Hirst DG and McKeown SR (1998) Increased expression of cytochrome P450 isoforms in mouse tumours and their role in the enhanced activation of the bioreductive drug, AQ4N. *Br J Cancer* **78**: 710

Patterson LH (1993) Rationale for the use of aliphatic N-oxides of cytotoxic anthraquinones as prodrug DNA-binding agents – a new class of bioredutive agent. *Cancer Metastas Rev* **12**: 119–134

Patterson LH and Raleigh SM (1998) Reductive metabolism: its application to prodrug activation. In: *Drug Metabolism Towards the Next Millennium*, Gooderham NJ (ed), pp. 72–79 IOS Press, Amsterdam

Patterson LH, Craven MR, Fisher GR and Teesdalespittle P (1994) Aliphatic amine N-oxides of DNA-binding agents as bioreductive drugs. *Oncol Res* **6**: 533–538

Patterson AV, Saunders MP, Chinje EC, Patterson LH and Stratford IJ (1998) Enzymology of tirapazamine metabolism: a review. *Anti-Cancer Drug Design* **13**: 541–573

Patterson LH, McKeown SR, Robson T, Gallagher R, Raleigh SM and Orr S (2000) Antiumour prodrug development using cytochrome P450 (CYP) mediated activation. *Anti-Cancer Drug Design* (in press)

Quinn PKM, Bibby MC, Cox JA and Crawford SM (1992) The influence of hydralazine on the vasculature, blood perfusion and chemosensitivity of MAC tumors. *Br J Cancer* **66**: 323–330

Raleigh SM, Wanogho E, McKeown SR, Burke MD and Patterson LH (1998) Involvement of human cytochromes P450 (CYP) in the reductive metabolism of AQ4N, a hypoxia activated anthraquinone di-N-Oxide prodrug. *Int J Radiat Biol Chem Physics* **42**: 763–767

Siim BG, Menke DR, Dorie MJ and Brown JM (1997) Tirapazamine-induced cytotoxicity and DNA damage in transplanted tumors: relationship to tumor hypoxia. *Cancer Res* **57**: 2922–2928

Smith PJ, Blunt NJ, Desnoyers R, Giles Y and Patterson LH (1997*a*) DNA topoisomerase II-dependent cytotoxicity of alkylaminoanthraquinones and their N-oxides. *Cancer Chemother Pharmacol* **39**: 455–461

Smith PJ, Desnoyers R, Blunt N, Giles Y, Patterson LH and Watson JV (1997*b*) Flow cytometric analysis and confocal imaging of anticancer alkylaminoanthraquinones and their N-oxides in intact human cells using 647-nm krypton laser excitation. *Cytometry* **27**: 43–53

Stratford IJ and Workman P (1998) Bioreductive drugs into the next millenium. *Anti-Cancer Drug Design* **13**: 519–528

Stratford IJ, Adams GE, Godden J, Howells N and Timpson N (1989) Induction of tumour hypoxia post-irradiation: A method for increasing the sensitizing efficiency of misonidazole and RSU 1069 in vivo. *Int J Radiat Biol* **55**: 411–422

Stratford IJ, Godden J, Howells N, Embling P and Adams GE (1987) Manipulation of tumour oxygenation by hydralazine increases the potency of bioreductive radiosensitizers and enhances the effect of melphalan in experimental tumours. *Radiat Res*, **2**: 737–742

© 2000 Cancer Research Campaign

Twentyman PR, Brown JM, Gray JW, Franko AJ, Scoles MA and Kallman RF (1980) A new mouse Tumor model system (RIF-1) for comparison of end-point studies. *J Natl Cancer Inst* **64**: 595–604

Wilson WR, Denny WA, Pullen SM, Thompson KM, Li AE, Patterson LH and Lee HH (1996) Tertiary amine N-oxides as bioreductive drugs: DACA N-oxide, nitracrine N-oxide and AQ4N. *Br J Cancer* **74**: S43–S47

Workman P and Stratford IJ (1993) The experimental development of bioreductive drugs and their role in cancer therapy. *Cancer Metastas Rev*, **12**: 73–82

Workman P, Balmain A, Hickman JA, McNally NJ, Mitchison NA, Pierrepoint CG, Raymond R, Rowlatt C, Stephens TC and Wallace J (1988) UKCCCR guidelines for the welfare of animals in experimental neoplasia. *Br J Cancer* **58**: 109

*© 2000 Cancer Research Campaign*
